# Nuclear pores enable sustained perinuclear calcium oscillations

**DOI:** 10.1186/s12918-016-0289-9

**Published:** 2016-07-22

**Authors:** Teresa Vaz Martins, Matthew J. Evans, Derin B. Wysham, Richard J. Morris

**Affiliations:** Computational & Systems Biology and Crop Genetics, John Innes Centre, Norwich Research Park, Norwich, UK; Mathematics Department, Wenatchee Valley College, Wenatchee, USA

**Keywords:** Calcium signalling, Nuclear pores, Fire-diffuse-fire

## Abstract

**Background:**

Calcium signalling relies on the flux of calcium ions across membranes yet how signals in different compartments are related remains unclear. In particular, similar calcium signals on both sides of the nuclear envelope have been reported and attributed to passive diffusion through nuclear pores. However, observed differing cytosolic and nucleosolic calcium signatures suggest that the signalling machinery in these compartments can act independently.

**Results:**

We adapt the fire-diffuse-fire model to investigate the generation of perinuclear calcium oscillations. We demonstrate that autonomous spatio-temporal calcium patterns are still possible in the presence of nuclear and cytosolic coupling via nuclear pores. The presence or absence of this autonomy is dependent upon the strength of the coupling and the maximum firing rate of an individual calcium channel. In all cases, coupling through the nuclear pores enables robust signalling with respect to changes in the diffusion constant.

**Conclusions:**

We show that contradictory interpretations of experimental data with respect to the autonomy of nuclear calcium oscillations can be reconciled within one model, with different observations being a consequence of varying nuclear pore permeabilities for calcium and refractory conditions of channels. Furthermore, our results provide an explanation for why calcium oscillations on both sides of the nuclear envelope may be beneficial for sustained perinuclear signaling.

**Electronic supplementary material:**

The online version of this article (doi:10.1186/s12918-016-0289-9) contains supplementary material, which is available to authorized users.

## Background

Changes in the concentration of free calcium ions (Ca^2+^) within cellular compartments [1–3] can act as a key information carrier in plants and animals [4–6]. Insights into how the different compartments may work together to generate specific spatiotemporal calcium patterns - the calcium signatures - could help understand the coordination of calcium regulated processes at the cellular level. In the nucleus, calcium regulates important functions, such as kinase activation [7], apoptosis [8], gene transcription [1, 9, 10] and neuron adaptation [11]. The autonomy of nuclear calcium signalling [6, 12, 13], however, remains controversial [14–19].

Nuclear calcium oscillations are often accompanied by cytosolic calcium changes and synchronised cytosolic/nuclear calcium oscillations have been recorded [20, 21]. Several studies have identified the cytosol as the source of nuclear calcium [22], an observation consistent with the nuclear envelope (NE) being permeated by pores whose diameter is large enough to allow the passage of small proteins [23]. Although experiments suggest that pores can become impermeable to ions in some conditions, the evidence is mixed [24–27] and it is unclear whether the free diffusion of small Ca^2+^ ions would be hindered [28]. Biological evidence [14] supports the view that a nuclear pore can adopt a conformation in which Ca^2+^ can freely diffuse through it, the debate being whether it can also adopt a calcium-impermeable conformation. The flux of calcium ions across membranes has been shown [29] to induce diverse effects on calcium oscillations.

However, the existence of calcium transients in isolated nuclei [30–32], significant delays and persistent gradients between cytosolic and nuclear transients [33], and the observation that different stimuli can selectively activate only one of the compartments [33] suggests that cytosolic and nucleosolic calcium levels are independently regulated. To generate independent signals, compartments would need to have their own calcium signalling machinery. Indeed, calcium release channels and pumps have been identified on both sides of the NE [14, 16, 20, 34–38], and the NE has been suggested to be a Ca^2+^ store [20, 39, 40]. Yet, free Ca^2+^ passage through permeable pores provides an additional source of calcium, and questions the independence of nuclear Ca^2+^ signalling.

The conflicting observations, i.e. synchronised oscillations versus compartment-specific transients, concerning the autonomy of nuclear Ca^2+^ signals may stem from the use of different cell systems and electrophysiological conditions. Furthermore, there are known limitations of experimental techniques used to quantify calcium levels, such as the spatio-temporal resolution of confocal imaging, or the different behaviour of calcium probes in the nucleus and cytosol [14]. Mathematical models [20, 41, 42] have been proposed to identify the minimal elements necessary to reproduce calcium nuclear transients in different scenarios. Thus, in parallel with experiments that showed calcium release in isolated nuclei of tobacco cells, Brière et al. [41] proposed a mechanism of Ca^2+^ release and decay based on a fully independent nuclear signalling machinery. This model accounted for isolated nuclei, but it is important to know the impact of pores under physiological conditions. Assuming that the nucleus did not have its own release machinery, a simplified model of cytosolic Ca^2+^ diffusion into the nucleus was shown to reproduce the differences between nuclear and cytosolic Ca^2+^ transients, observed in ventricular myocites [42]. Strengthening the idea that simple diffusion would not result in similar signatures, a model of symbiotic calcium signaling of legumes [20] showed that diffusion across pores cannot reproduce the near simultaneous equivalent nuclear and cytosolic calcium patterns, measured around the perinuclear region. They conclude [20] that calcium is probably released from either side of the nuclear envelope, leaving open the explanation for their coordinated release.

**Fig. 1:**
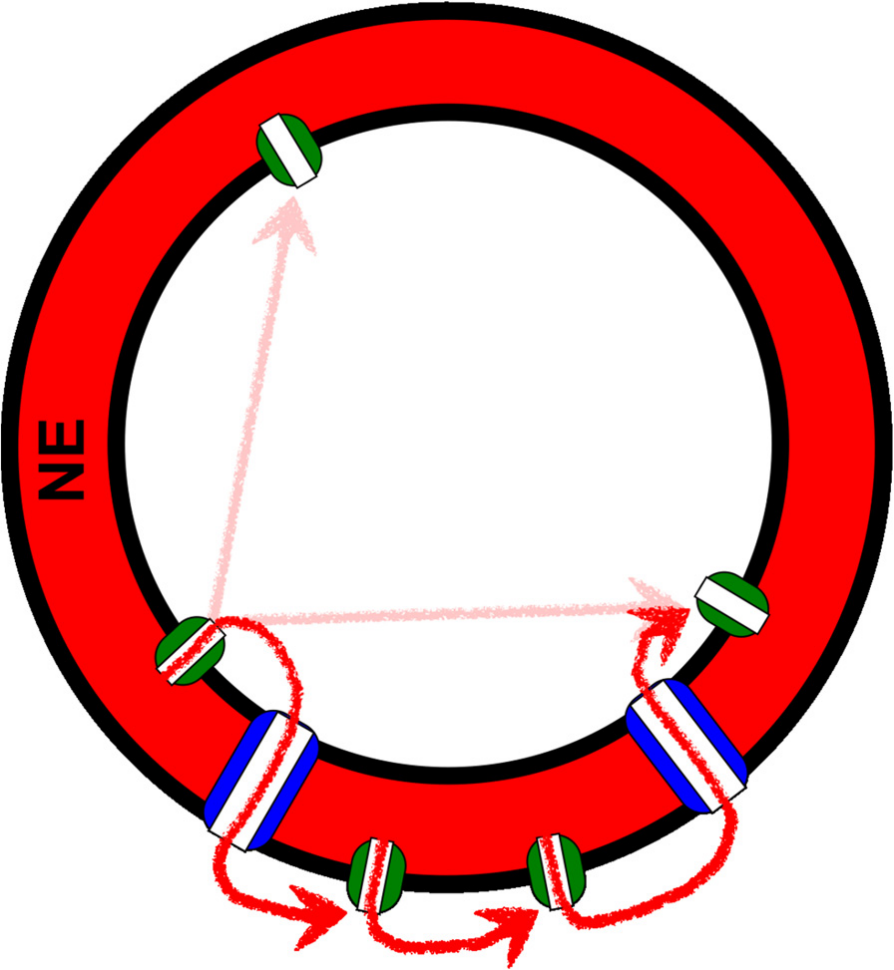
The basic model components and possible paths to channel activation. Ca^2+^ is released from the nuclear envelope (NE) store and the effect of pumps is modelled as a uniform uptake into the NE. Channels are shown in *green* and pores in *dark blue*. In our model, CICR activation can fail for several reasons such as a low diffusion coefficient or large inter-channel distances. If the nucleosol has channels that are too far apart, Ca^2+^ cannot spread far and activate directly another channel (*pink path*). The red curve illustrates an alternative path to activation that exploits the more favourable cytosolic propagation conditions, with closer cytosolic channels

Here we use mathematical modelling to identify conditions that, from the interaction between the same basic components, give rise to different behaviour. The Fire-Diffuse-Fire (FDF) model captures the contribution of spatially localised channels to calcium signalling in a simple way [43–49], assuming that upon release calcium diffuses to activate further channels according to the calcium-induced-calcium-release (CICR) process (Methods). This is a common channel gating mechanism; the IP_3_R channels that have been found on both sides of the NE in animals regulate Ca^2+^ release in response to IP_3_ and calcium binding to receptor sites. We model Ca^2+^ diffusion over the spherical surface representing the NE and through nuclear pores that connect the inner and outer nuclear membranes, placing channels on both sides of the NE. The system of pores and channels is sketched in Fig. 1, which illustrates possible paths that Ca^2+^ can follow to activate channels. We have not considered calcium coming from farther cytosolic sources, which would probably arrive at the nuclear periphery in negligible concentrations [14]. The interaction between calcium released by two sets of channels in close proximity on either side of the NE constitutes the biggest challenge to the autonomy of nuclear calcium signalling. We show that the coupling between the nucleus and cytosol is a key ingredient for the robustness of perinuclear Ca^2+^ oscillations with respect to changes in the diffusion constant of Ca^2+^, but that this coupling may nevertheless allow for autonomous Ca^2+^ signatures on either side of the NE.

## Results and discussion

### Nuclear pores can make the nuclear envelope transparent to Ca^2+^

**Fig. 2:**
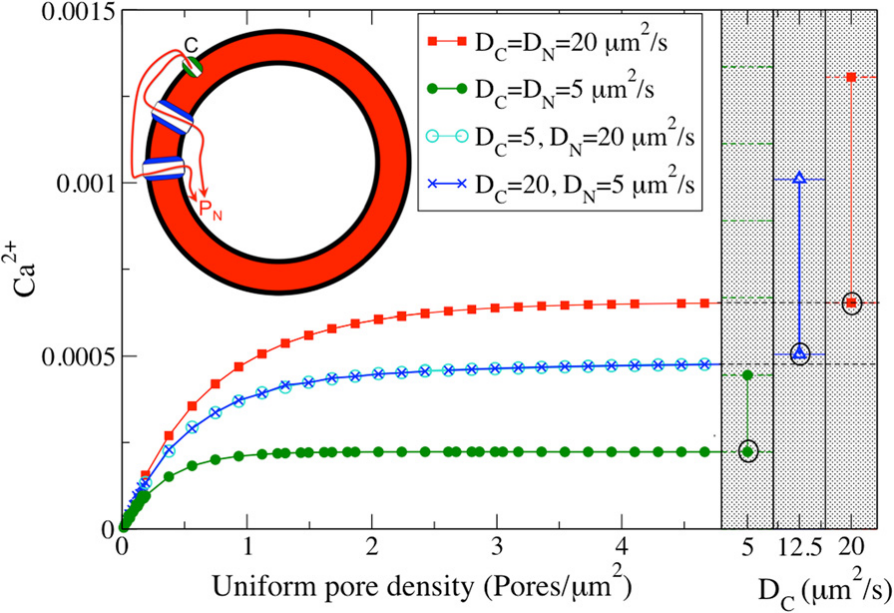
The amount of Ca^2+^ transmitted by a firing channel increases with the number of pores for evenly spaced pores, until it saturates. White panel (*left*): maximum Ca^2+^ concentration measured at a reference point *P*_N_, in response to single channel *C* release. *P*_N_ and *C* are located in different sides of the NE, and example Ca^2+^ paths are depicted in red. The pores are distributed evenly across the surface. In general, a larger transmission is achieved for a larger number of pores. For the numbers and positions of pores represented, larger diffusion constants lead to larger transmissions. In the firing event, the channel releases *σ*=2.0× 10^−20^ mol of Ca^2+^. Ca^2+^ levels are given as a fraction of the released amount *σ*. For comparison, in the shaded right panels we consider a point at the same distance from *C* as *P*_N_, but on the same side as the release channel on a NE without pores. We consider three pairs of values for the diffusion coefficient *D*, and for each *D*, the larger value corresponds to the same release amount as in the non-shaded panel, *σ*=2.0×10^−20^ mol and the lowest to half that value, *σ*=1.0×10^−20^ mol. As in the other panel, we also rescale the calcium units by dividing by 20.0. Other parameter: *α*=0.15

Different species and cellular stages can have different pore numbers and distributions [50–56], with pore densities varying between 1 and 60 pores /*μ*m^2^ [14]. We accounted for this variability as well as possible gating phenomena [28, 57] by varying the number and position of calcium-permeable pores. To investigate the influence of pore number on transmission across the NE, we asked how much Ca^2+^ spreads from a channel, *C*, firing once on the cytosolic side of the NE to a point on the nucleosolic side of the NE, *P*_N_, Fig. 2. We consider distances, *C* to *P*_N_, that are larger than Ca^2+^ micro-domains formed near firing calcium channels. We measured the maximum amount of Ca^2+^ that reaches *P*_N_ relative to the release quantity from *C*.

We observe in Fig. 2 that Ca^2+^ transmission increases with pore density before saturating at relatively low pore densities of about 2 pores /*μ*m^2^. The addition of pores does not increase the transmission further. To understand the meaning of this saturation, we asked what would happen if *P*_N_ was on the same side as *C*. If there were no pores present then, as illustrated in the grey region of Fig. 2, the maximum concentration of Ca^2+^ measured at *P*_N_ would be twice the value measured before. This happens if channel *C* had released the same amount of Ca^2+^ as before. The circled values in the grey region of Fig. 2 correspond to maximum concentration measured at *P*_N_ when *C* releases half as much Ca^2+^. For the cases where the diffusion constants on either side of the NE are equal, we see that these circled values coincide with the saturation values. This illustrates that when we have a saturation pore density half the Ca^2+^ released by a channel is passing through the pores to the other side of the NE. This is equivalent to diffusion of a substance in a half space, compared to diffusion without the barrier, in a full space. It confirms that as far as diffusing Ca^2+^ is concerned, beyond a certain pore density the NE provides no barrier to passage between the two sides. We refer to this as the NE becoming transparent.

Slower diffusion hinders propagation over long distances by giving pumps time to take significant amounts of Ca^2+^ back into the NE. This limits the maximum amount of Ca^2+^ that can be transmitted for a smaller diffusion constant *D* (blue line, Fig. 2). However, when the NE becomes transparent, the two surfaces combine their Ca^2+^ propagation abilities to behave as a single surface with an intermediate diffusion constant for Ca^2+^. This can be seen in Fig. 2 by comparing the transmission between surfaces for D=5 and 20 *μ*m^2^/s, with the transmission on a single surface with D=12.5 *μ*m^2^/s. As shown in Fig. 2, the increased diffusion efficiency that results from access to a surface with larger *D* may even compensate for the loss that results from sharing the released Ca^2+^ with another compartment.

### Pores increase transmission by creating diffusion paths between channels on different sides of the nuclear envelope

Even with a large pore diameter of 0.029 *μ*m, the pore density at transmission saturation accounts for only 0.8 *%* of the nuclear surface (for a nucleus of radius R=8.0 *μ*m). This led us to look more carefully into the actual contribution of individual pores to transmission. The spatial distribution of pores determines the characteristics of the Ca^2+^ diffusion paths between calcium release channels, which will be relevant for the CICR activation.

**Fig. 3:**
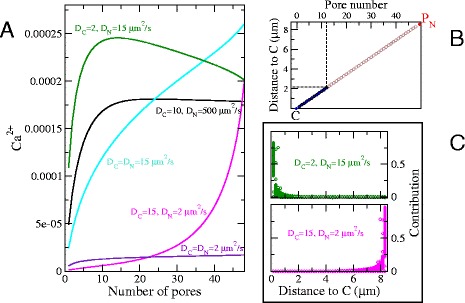
Pores far from a channel in a region with a low diffusion constant have a negligible contribution to Ca^2+^ transmission across the NE. Pores are distributed on the great arc between a channel *C* on the ONM and a measurement location *P*_*N*_ on the nuclear side. Ca^2+^ levels are given as a fraction of the released amount. The panel **b** shows the pores arrangement between the channel *C* and the point *P*_*N*_: each additional pore occupies the following place on a queue that starts close to *C*. Panel **a** the transmitted Ca^2+^ as a function of the number of pores. Panels **c** For the case of 48 pores, their contribution as a function of their distance to the channel *C*. The different points along a vertical line correspond to different times. Pores far from the site located on the side with a low diffusion constant have a negligible or even slightly negative contribution. We sum the contributions over time of an individual pore, up until the time where the calcium concentration at *P*_*N*_ reaches its maximum value. The results in panels **c** show the fraction contributed by a pore, in relation to the total contribution by all the pores *α*=0.15

We define the contribution of a pore as the increase or decrease of the local Ca^2+^ concentration that results from Ca^2+^ diffusing from a given pore (Methods). In Fig. 3 we investigate the individual contribution of pores to the transmission from channel *C* to the measurement site in the other compartment, *P*_N_, with pores placed along a line connecting the two, Fig. 3b. The channel *C*, fires once to release Ca^2+^, which then diffuses. Ca^2+^ can diffuse through pores, cross the nuclear envelope and increase or decrease the concentration measured at *P*_N_. The results of Fig. 3c were obtained by counting at each time step how much Ca^2+^ reaches point *P*_N_ from a given pore. We summed the contribution of a pore until the Ca^2+^ concentration reaches its maximum value, and then we divide by the total contribution from all pores to obtain the fraction corresponding to an individual pore.

In Fig. 3a we show the transmission across the NE from *C* to *P*_N_ for varying pore numbers along the line between *C* and *P*_N_ and for different diffusion constants. We note that the effect of the spatial configuration of the pores depends on the relative values of the diffusion constant *D* for Ca^2+^ on both sides of the NE. Ca^2+^ does not propagate over long distances in highly buffered surfaces, those with low *D* (Methods).

As seen in Fig. 3c, the contribution of pores to transmission is higher the closer they are to the site, either *C* or *P*_N_, on the surface with a lower diffusion constant. When the side with *C* has a lower *D*, the dominant contribution is from pores near to *C*, and pores near *P*_N_ have a negligible contribution. When the side with *P*_N_ has a lower *D*, the situation is reversed and the dominant contribution comes from pores close to *P*_N_. This demonstrates that a relatively small number of pores clustered around channels can achieve an effective transparency for Ca^2+^ of the NE.

Moreover, a high pore density may hinder transmission, as shown by the green line in Fig. 3a, when D _*C*_=2, D _*N*_=15 *μ*m^2^/s. This surprising effect happens when Ca^2+^ enters a nucleus with larger diffusion constant for Ca^2+^. Under these conditions Ca^2+^ spreads faster in the nucleus and nuclear Ca^2+^ concentration will be higher at some distance from the entry pore. If there is a pore at that location, it will allow Ca^2+^ to diffuse through and reenter the cytosol to restore a concentration balance, thus lowering nuclear Ca^2+^ levels.

We conclude that the distribution of pores relative to the channels may be as, if not more, important than their number. Short and numerous paths facilitate the CICR activation, whilst the addition of pores that do not shorten the length of the diffusion path do not contribute significantly to the overall transmission. Biological evidence shows that in several systems pores are not evenly spaced over the surface of the NE [50, 51, 55, 56], and that at least in fungi they can be mobile [58, 59]. Further experiments are needed to discover the constraints behind the adoption of a particular distribution, whether it serves to optimise nuclear transport, and in particular how it relates to the position of the Ca^2+^ channels and the characteristics of the environment in which the signalling process takes place. Our modelling suggests that it may be advantageous in terms of signalling if the clustering of pores was coordinated with the location of the Ca^2+^ release channels. Pores and release channels on both sides of the NE give rise to a Ca^2+^ diffusion path that is robust against variations in the diffusion coefficient *D*, since in regions of low *D*, Ca^2+^ may take a shortcut via the other side of the NE with larger *D*, Additional file 1.

### Autonomous calcium signatures can coexist despite interdependence

Free Ca^2+^ diffusion across pores couples the Ca^2+^ firing events in the cytoplasm and nucleopasm, offering an increased robustness to the signalling process. However, experimental observations indicate that either similar or specific spatiotemporal Ca^2+^ patterns in the two compartments can occur. We asked whether coupling the oscillations can achieve robustness whilst maintaining oscillations of different frequencies on either side of the NE.

**Fig. 4:**
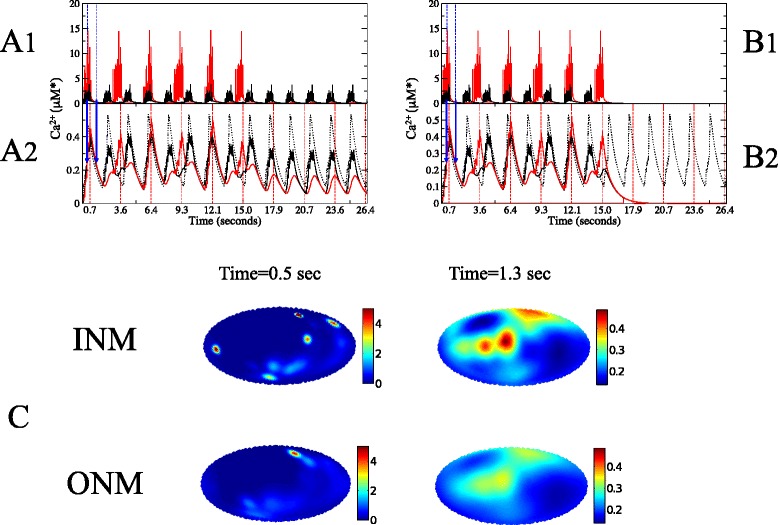
The hierarchical scale of the Ca^2+^ signature, when a porous membrane allows oscillations to be sustained on both sides of the NE. An interior channel fires first. The colours red and black refer to INM and ONM, respectively. The diffusion constant on the inside is D _*N*_=5 *μ*m^2^/s, and on the outside is D _*C*_=20 *μ*m^2^/s. The inner and outer membrane are coupled by 1500 uniformly randomly distributed pores, corresponding to a pore density of 1.9 pores/ *μ*m^2^. Panels **a** and **b**, depict the temporal evolution of the Ca^2+^ concentration: panels **a**1 and **b**1 show the Ca^2+^ levels surrounding channels, averaged over the 24 channels, while panels **a**2 and **b**2 shows levels averaged over 5000 points evenly distributed on the sphere [74]. The x-axis intervals correspond to the refractory period of the INM channels: 2.86 seconds. The dotted oscillating lines represent the average global Ca^2+^ concentration on an isolated ONM. On the panels **a**, the INM channels were shut at t=15 s, leaving the ONM channels unaffected. On the panels **b**, the ONM channels were shut at t=15 s, leading to the interruption of Ca^2+^ release by the INM channels. The panels **c**, are an atlas view of the inner surface (top) and the outer surface (bottom) when the inner and outer global oscillations coincide [75], at t=0.5 seconds (left) and t=1.3 seconds (right). These times are signalled by the vertical blue arrows in panels **a**2 and **b**2. In these two moments the global concentration is approximately the same and yet the spatial profiles are very different. For presentation, the upper limits of the left atlas are capped at 5 *μ*M*. The upward phase of the cycle (t=0.5 s) occurs when multiple channels are firing, resulting in a heterogeneous spatial profile with clear microdomains, while the downward phase (t=1.3 s) happens after firing events when Ca^2+^ is diffusing away from the channels. *α*=0.15, k _*s*_=1.0 s ^−1^. The results don’t depend on the side of the initial firing channel. For the meaning of the M* units, see first subsection in Methods

In this section we show that under certain conditions distinct oscillation frequencies can occur in the different compartments despite the coupling of channel activation that occurs in the presence of pores. We considered a nucleus with 24 randomly placed channels on each side of the nucleus, and studied the generation of signals in the two compartments. In Fig. 4a and b we illustrate the temporal evolution of Ca^2+^ concentration, both on the nuclear (red lines) and cytosolic (black lines) sides of the NE. We consider a pore density of 2 pores/ *μ*m^2^ so the results in Fig. 4 refer to a transparent NE.

The cytosolic side of the NE is taken as having a larger diffusion constant, *D*, which permits cytosolic Ca^2+^ oscillations in the absence of pores. We demonstrate this by shutting the channels on one side of the NE after 15 seconds, to see the effects on the other side. Inactivating the nucleosolic channels leaves cytosolic calcium release unaffected, Fig. 4a1. In contrast, the nuclear Ca^2+^ oscillations can not be sustained without cytosolic influx. As illustrated in Fig. 4b1, if the cytosolic channels are shut down, the nucleosolic calcium oscillations are also aborted, indicating that it is the cytoplasmic channels that are driving this nuclear oscillation.

Once a channel releases Ca^2+^ it enters a refractory period during which it will not fire (Methods), thereby defining a maximum firing rate. Within the Fire-Diffuse-Fire framework, the refractory period is chosen somewhat ad hoc, often to match experimental values [46] or to investigate hypothetical scenarios. Here we investigate the impact of coupling oscillations on either side of the NE that may have different frequencies. Therefore we have chosen refractory periods that depend primarily on the properties of the compartment and not on their coupling: the compartments with lower *D* have a larger refractory period (Methods). Under these conditions, Fig. 4a & b shows that an integrated perinuclear signalling machinery, composed of nuclear and cytosolic channels with independent refractory periods coupled by a transparent NE, will result in an average nuclear Ca^2+^ concentration that oscillates with an autonomous frequency, but requires the input of cytosolic Ca^2+^.

As seen in Fig. 4a1 and b1, the nucleosolic channels fire periodically until we inactivate the cytosolic channels. However, the larger refractory period of the nucleosolic channels prevents them from firing as often as the cytosolic channels fire. Ca^2+^ influx into the nucleus increases when the cytosolic channels fire. This influx is large enough to maintain local nuclear Ca^2+^ levels above the channels’ activation threshold, even between cytosolic release cycles. This allows the nucleosolic channels to fire immediately as soon as they emerge from their refractory period, even if the cytosolic channels are resting at the time. Thus, in the case of a transparent NE, the global Ca^2+^ frequency coincides with the individual refractory periods, which are, as mentioned, taken to be a characteristic of each compartment. For a discussion of the effect of weaker coupling, and how alternative implementations of the refractory period may lead to synchronisation, see Additional file 2.

Influx through pores is responsible for the smaller oscillation peaks that can be seen in Fig. 4a2 and b2 as an increase in nuclear Ca^2+^ that coincides with the cytosolic increase. When the nuclear calcium channels are shut, these peaks are all that remain, making the cytosolic and nuclear calcium oscillations oscillate with the same frequency, but different amplitudes. The intermediate case of channels firing in an uncoordinated manner is less clear, as shown in Additional file 2.

We conclude that a transparent NE still allows nuclear and cytosolic Ca^2+^ signalling to exhibit autonomous frequencies, even if the oscillations are not independent, so long as channel refractory periods in the two compartments are different. By contrast, given that the time to transmit the first Ca^2+^ wave is negligible (see Fig. 4a1 and b1), if the refractory period was the same on both sides of the NE then nucleosolic and cytosolic Ca^2+^ oscillations could be synchronised, as observed for instance during plant symbiosis [20] (see Additional file 2). It has been argued that Ca^2+^ signatures encode information about the stimuli and determine the elicited response [12, 13, 60]. In cases for which nuclear and cytosolic Ca^2+^ signals perform different roles, different spatiotemporal patterns in these compartments may be essential. Yet even under these circumstances, the flux of calcium through pores can play an essential role in maintaining the oscillations in both compartments.

### Global calcium oscillations can mask details of microdomain dynamics

The frequency of oscillations is an important characteristic to differentiate between signals but Ca^2+^ spatio-temporal patterns can be distinguished at various levels. In addition to looking at surface averaged signals, local spatio-temporal patterns may also be important.

The release of Ca^2+^ from a cytosolic channel generates a small region of high Ca^2+^ concentration near the channel, called a Ca^2+^ micro-domain [61]. Even under conditions of transparency the cytosolic micro-domain is not directly reproduced in the nucleosol, instead nuclear Ca^2+^ levels accumulate and spread gradually. However, if Ca^2+^ flow triggers the firing of a nucleosolic channel, then nuclear microdomains are also observed. These are shown in Fig. 4b as atlas views of the nuclear surfaces. We can observe nearly symmetric Ca^2+^ micro-domains on both sides of the NE which are consistent with the activation path from a cytosolic channel, through pores, to the nearest nucleosolic channels.

Figure 4 compares Ca^2+^ levels surrounding release sites (Fig. 4a1 and b1) with the average over the membranes (Fig. 4a2 and b2), showing that while the amplitude of the global oscillations is similar on both sides, it is considerably more elevated in the neighbourhood of the inner channels (red, Fig. 4a1 and b1). Therefore, although inner and outer channels released the same amount of Ca^2+^, the microdomains of Ca^2+^ concentration persisted for a longer time and were confined to a smaller region on the side with a lower diffusion constant. The spatio-temporal patterns of Ca^2+^ transients can be studied by imaging techniques [62]. However, during the measurement of Ca^2+^ transients in living systems, the nucleus is moving around and slightly changing shape. Since it is challenging to measure calcium transients at the scale of a single channel, experimental measures often refer to various larger spatial scales such as whole nuclear averages, which need to be carefully interpreted. The distinct nuclear Ca^2+^ signature with very high and persistently localised microdomains could not appear unless supported by oscillations on the other side of the membrane. By contrast, the global oscillations (second row) have similar amplitudes, since Ca^2+^ microdomains with very different amplitudes and spatial extent can result in the same global Ca^2+^ level, as the comparison of the spatial profiles represented in Fig. 4a and b show.

## Conclusions

Experimental data shows a variety of similarities but also differences between cytosolic and nuclear Ca^2+^ patterns [63–68]. In particular, there have been disagreement about the existence and independence of nuclear Ca^2+^ oscillations. Here we show that within one framework we can obtain 1) oscillations on only one side of the NE for different diffusion constants and little flux through the nuclear pores, 2) synchronised Ca^2+^ signals in cases where the nuclear pores transmit sufficient Ca^2+^ between different sides of the NE if the channels have the same refractory period, 3) different oscillations when the NE becomes transparent to Ca^2+^and when different conditions on either side of the NE lead to different refractory periods. Different conditions could be caused by different buffering capacity for Ca^2+^ [69]. Furthermore, we observe synchronised oscillations with different amplitudes when only channels on one side fire in a coordinated manner. This demonstrates how different observations can be reconciled if the oscillations on both sides of the nuclear envelope are connected by passive diffusion through nuclear pores. We have shown that the presence of calcium release channels on both sides of the nuclear envelope reinforces nuclear and cytosolic Ca^2+^ oscillations, which may be important for sustaining oscillations over longer periods in varying conditions.

We demonstrated that free diffusion through the NE allows different cytosolic and nuclear Ca^2+^ oscillation patterns to be maintained, even when the oscillations are coupled via diffusion through the nuclear pores. This coupling may be essential to sustain oscillations. This result reveals a new role for pores, as elements that increase the robustness of the channels’ activation by CICR, against variations in the diffusion coefficient or buffering conditions of the inner and outer nuclear membranes. Therefore, instead of the Ca^2+^ flowing from the cytosol through pores being a significant fraction of the nuclear Ca^2+^ transients, it may simply provide access to an alternative path of diffusion in the fire-diffuse-fire process.

Our models reveal that the permeability of the nuclear envelope is determined not only by the number and size of pores, but also by their distribution and the characteristics of the environments they connect, such that a large number of pores does not necessarily translate into a larger permeability.

We have assumed that channel gating depends on Ca^2+^ binding, which is supported by the identification in some systems of IP_3_R channels on both sides of the NE. Our results are valid also for indirect forms of calcium-induced calcium release, e.g., if Ca^2+^ activates a cation channel that regulates a voltage-gated calcium channel [69, 70]. Nevertheless, channels and their activation mechanisms are yet to be fully identified in several systems, and gating other than CICR is possible. It is plausible that mechanically or temperature activated channels would sustain independent oscillations [32, 41], or that voltage-gated channels would be simultaneously activated by the polarisation of the nuclear membrane. However, a more versatile communication is possible between CICR-activated channels, where oscillations may be interdependent with autonomous patterns. Therefore, we suggest that the comparison between nuclear and cytosolic Ca^2+^ oscillations patterns offers a clue to the channels activation mechanism.

The refractory period has an important influence on the overall Ca^2+^ signals observed. While we know channels enter a refractory period during signalling, what mechanism controls the length of the refractory period during biological signalling processes is still unclear [71]. Given these numerous dependencies it is quite possible for different maximum firing rates to arise in some situations and not in others. Our results indicate that as the mechanisms responsible for the duration of a channel’s refractory period become better known in different systems, they may lead to an understanding of the variety of differences and similarities between nuclear and cytosolic Ca^2+^ signatures.

Next steps include extending our model to a three-dimensional implementation to investigate the role of morphology in determining different nuclear Ca^2+^ signatures [72], as well as to ascertain which parameters are key for signal propagation and robustness [73].

## Methods

### Fire-Diffuse-Fire over a nuclear membrane.

We adapt the fire-diffuse-fire model [43] with pumps [45] and pores [20] to include channels on both sides of the nuclear membrane. The spread of Ca^2+^ is reduced by the uptake into the nuclear envelope (NE) store, which we modelled as a uniform pump rate across the NE that depends linearly on the Ca^2+^ concentration. On the surface of a NE without pores, the Ca^2+^ concentration *c*=*c*(**r**,*t*) evolves with time as:

1 $$ \frac{d c(\mathbf{r},t)}{d t} = D\nabla^{2} c(\mathbf{r},t) - k_{\mathrm{s}} c(\mathbf{r},t) + \sum\limits_{i=1}^{N} f_{i}(t) \delta(\mathbf{r}-\mathbf{r}_{i}),  $$

where *D* is the diffusion constant and *k*_s_ is the rate at which Ca^2+^ is pumped back into the store (the nuclear envelope, NE). For comparability of units, we have assumed that the relevant domain for the concentration, *c*(**r**,*t*), is a thin layer, however, we highlight the fact that all the calculations are performed on a surface by using the units M ^∗^ to indicate that it’s is not strictly per volume.

We consider the interaction between N channels located at positions **r**_*i*_ on the same side of the NE. The calcium release channels are treated as point sources, which are expressed as *δ*-functions. We explore the saltatory regime [43] of the FDF model, assuming instantaneous opening/closing of channels at firing times ${t^{i}_{k}}$ for each channel *i*=1,…,*N* and *k*=1,2,…

2 $$ f_{i}(t) = \sigma \delta \left(t-{t_{k}^{i}} \right),  $$

where *σ* is the amount released by a channel in a single firing event. To determine the firing times according to the CICR mechanism, we assume that the channel will fire if the local Ca^2+^ concentration, computed at the channels’ coordinates, is greater than a given threshold concentration, *c*_th_.

Ca^2+^ is released from multiple stores such as the ER, mitochondria or possibly the nucleoplasmic reticulum, and propagates in complicated geometries and crowded environments. However, to model the influence of pores on nuclear Ca^2+^ signalling, we note that the Ca^2+^ spreading from non-perinuclear regions would arrive at the nucleus in negligible proportions [14] and can be ignored. Therefore, we focus on the interface between the inner and outer nuclear membrane, for which the two-dimensional solution of the diffusion equation with no boundaries is a good approximation (see Additional file 3 for a comparison). At time *t* the Ca^2+^ concentration at a location **r**_*j*_ at the surface is:

3 $$ c_{i/o}(\mathbf{r}_{j},t) = \sigma\sum_{k,i}\frac{1}{4 \pi D \left(t-{t_{k}^{i}}\right)} e^{-\Delta{r}_{ij}^{2}/\left[4 D \left(t-{t_{k}^{i}}\right)\right]-k_{s} \left(t-{t_{k}^{i}} \right)}  $$

where *c*_*o*_(**r**_*j*_,*t*) and *c*_*i*_(**r**_*j*_,*t*) are the Ca^2+^ concentrations on the outer and inner sides of the nuclear envelope. Equation 3 is the solution of the diffusion equation for a two dimensional space with no boundaries, but we constrained Ca^2+^ to move across the surface of the sphere representing the nucleus. The position **r** is expressed in spherical coordinates as **r**=(*R* sin*θ* cos*ϕ*,*R* sin*θ* sin*ϕ*,*R* cos*θ*), where *θ* is the polar angle, *ϕ* the azimuthal angle and *R* is the radius of the nucleus. In this case, the shortest distance *Δr*_*ij*_ between a source **r**_*i*_ and any point **r**_*j*_ is the length of the great circle arc that connects them, given by

4 $$ {}\Delta {r}_{ij}=R\arccos\left(\cos\theta_{i}\cos\theta_{j}+\sin\theta_{i}\sin\theta_{j}\cos(\phi_{i}-\phi_{j})\right).  $$

Parameters: *N*=24 channels (for Fig. 4), radius of nucleus *R* = 8.0 *μ*m.

### Conditions for signalling propagation on an isolated membrane

The CICR activation of calcium channels by diffusion may fail by changing parameters such as the inter-channel distance, the number of channels, the value of the uptake rate *k*_*s*_, or the diffusion coefficient, *D* [43–45].

For a given activation threshold *c*_th_, we want to know if there is a time *t* for which the calcium concentration at a channel location, **r**_*j*_, is enough to activate the channel, that is, if *c*_*i*/*o*_(**r**_*j*_,*t*)>*c*_th_. So we find the time *t* that satisfies:

5 $$  \max_{t}\frac{1}{4\pi Dt}e^{-\frac{\Delta\bar{r}^{2}}{ 4 Dt}-k_{s}t} \geq\frac{c_{\text{th}}}{\sigma}.  $$

Once choices of the average inter-channel distance $\Delta \bar {r}$ and release amount *σ* have been made, the condition for the critical surface for propagation in terms of the parameters *k*_*s*_ and *D* becomes

6 $$ {}\begin{aligned} \frac{k_{s}}{2\pi \! D \! \left(\!\!\sqrt{1 \! +\! k_{s}\Delta\bar{r}^{2}/D}\,-\,1\! \right)}e^{-\frac{k_{s}\Delta\bar{r}^{2}}{ 2 D \left(\!\sqrt{1+k_{s}\Delta\bar{r}^{2}/D}-1 \right)} -\! \left(\!\!\sqrt{1+k_{s}\Delta\bar{r}^{2}/D}-1 \!\right)/2}\!\! \geq \!\frac{c_{\text{th}}}{\sigma}. \end{aligned}  $$

For a given $\Delta \bar {r}$ and release amount *σ*, the activation requirement is fulfilled when the uptake rate is not too large, or the diffusion constant not too small. A graphical representation of the parameter choices for which traveling wave solutions exist appears in Additional file 4. In particular, in our 2D approximation the sink - pumping rate - is less efficient when *D* is larger. Faster pumps increase the risk of propagation failure unless *D* is large enough.

Characteristics of the signals, such as frequency or amplitude, are affected by various interchangeable parameters. Our illustrative examples use the diffusion coefficient *D* as the parameter to distinguish the Ca^2+^ propagation abilities on the inner and outer nuclear membrane, but the analogous results are expected for other choices that lead to either propagation failure or a Ca^2+^ wave. Throughout the paper and for simplicity, we have assumed a larger diffusion constant *D* on the cytosolic side of the NE but analogous conclusions hold if we reverse the diffusion constant ratio between different sides of the NE.

Unless otherwise stated in the figure legend, the results refer to the following parameters throughout the paper: firing threshold *c*_th_=0.0425 *μ*M*, amount released by a single release event *σ*=2.0×10^−20^ mol.

### Coupling the inner and outer nuclear membrane: pores as additional calcium sources

We couple the inner and outer nuclear membranes through the inclusion of pores, which we treat as point sources [20]. This adds additional source terms to the diffusion equation (Eq. 1),

7 $$  \sum\limits_{k=1}^{n_{p}}\Omega_{k}(t)\delta(\mathbf{r}-\mathbf{r_{k}}),  $$

where the summation is over all the *n*_*p*_ nuclear pores at positions **r**_*k*_, *k*=1,…,*n*_*p*_, having a flux through them of *Ω*_*k*_(*t*). We assume the pores are either closed, in which case *Ω*_*k*_(*t*)=0, or open allowing Ca^2+^ to passively diffuse through them according.

According to Fick’s law of diffusion, the flux across pores will depend on the calcium concentration gradient between the nucleus and the cytosol, which can be approximated by a difference formula at each time step (of size *Δt*). We assume that beyond their geometry, the pores pose no additional obstacle to diffusion. The thickness of the nuclear envelope, *Δm*, the diffusion constant for Ca^2+^ in the pore, *D*_*P*_, and its diameter, *p*_*d*_, determine the size of the pulse, given by

8 $$  \Omega_{k}(t) = -D_{P}/\Delta m (c^{*}_{o}(\mathbf{r}_{k},t)-c^{*}_{i}(\mathbf{r}_{k}, t)) (p_{d}/2)^{2} \pi \Delta t.  $$

We had, Eq. 3, the solution for diffusion along the surface of the nucleus, but to account for pores we are implicitly assuming diffusion along a direction perpendicular to that surface. For the two dimensional approximation to be justified, the underlying assumption is that the similar differences in concentrations would hold for a 3 dimensional space. Therefore, in the formula 8, we use for the volumetric nuclear and cytosolic concentrations differences, $(c^{*}_{o}(\mathbf {r}_{k},t)-c^{*}_{i}(\mathbf {r}_{k}, t))$, the same numerical values that we determined (by Eq.3), for the surface concentrations, (*c*_*o*_(**r**_*k*_,*t*)−*c*_*i*_(**r**_*k*_,*t*)).

At each time step, the flux *Ω* can therefore take negative and positive values depending on the relative concentration on the inner and outer nuclear membranes. The overall Ca^2+^ concentration, $c^{\dagger }_{i/o}(\mathbf {r}_{j},t)$, results from the joint contribution of calcium emanating from channels and from pores,

9 $$ {6pt}{}\begin{aligned} c^{\dagger}_{i/o}(\mathbf{r}_{j},t) &=c_{i/o}(\mathbf{r}_{j},t) \\ & \quad+ \sum\limits_{k=0}^{n_{p}} \sum\limits_{\tau_{i}}\frac{\Omega_{k}(t)}{4 \pi D (t-\tau_{i})} e^{-\Delta\mathbf{r}_{jk}^{2}/\left[4 D (t-\tau_{i})\right]-k_{s} (t-\tau_{i})} \end{aligned}  $$

where *c*_*i*_/*o*(**r**_*j*_,*t*)is the contribution from the channels on the same side of the NE, as before (Eq. 3), and the *τ*_*i*_ summation is over all the timesteps since the start of the simulation.

Using this approach, we investigate several pore distributions exploring clustering, random uniform distributions or approximations of an even-spaced distribution like the golden section spiral method [74]. For the projection of the sphere on an ellipse, we used the area-preserving Mollweide projection [75].

Parameters: pore diameter *p*_*d*_=0.029 *μ*m, thickness of the nuclear envelope △m=0.01 *μ*m, diffusion constant for Ca^2+^ through a pore *D*_*P*_=20 *μ*m^2^/s.

### From the single refractory period to oscillation frequencies

As reviewed in the Introduction, persistent gradients between nuclear and cytosolic Ca^2+^ signatures could indicate that the nucleus independently generates its own Ca^2+^ signals. We revisit this assumption by asking if coupling between oscillations has advantages to perinuclear Ca^2+^ signalling, without necessarily imposing the same oscillation frequency.

To answer this question, we first need to define an *autonomous frequency*, specific to the nucleus or cytosol. In the context of the FDF model, this can be done by imposing an interval of inactivity between successive firings of an individual channel. However, the original FDF models [43, 44] don’t provide any guidelines for its duration, and later adaptations [45, 46] have chosen values to match experimental magnitudes, without specifying a clear generating mechanism.

Indeed, the multiple mechanisms that underlie the duration of a refractory period are still unknown [71]. Many channels close due to inhibition of their gates at high calcium concentration [44], but then remain in a refractory state much longer than the time taken to fall back below this inhibitory level. This could represent the time taken to refill the local Ca^2+^ store [71], be dependent on the local concentration of cytoplasmic Ca^2+^ or other ions and signalling molecules, such as IP_3_R, or various other feedbacks of the signalling pathways that Ca^2+^ participates in.

In the main text, we investigate what happens when nucleosolic and cytosolic channels are maximally coupled by a transparent NE, but their refractory periods are independent. For a concrete choice, we follow [20], and equate its duration to the time elapsed until the Ca^2+^ that the channel itself had released has dropped below a certain fraction, *α*, of the activation threshold *c*_th_. Since this concentration does not include the contribution of the other channels and pores, it leads to a refractory period that is a characteristic of each compartment. Channels in a medium of low diffusion constant will take a longer time to see local Ca^2+^ drop, resulting in larger minimum resting periods.

Then, the observed nuclear Ca^2+^ oscillation frequency will coincide with the individual (and independent) channels’ refractory period as long as two conditions are fulfilled. First, coupling must be strong enough to allow the channels to fire at the maximum rate imposed by their refractory period. Secondly, nuclear channels should fire in unison, so their joint contribution to global Ca^2+^ levels is much larger than the pores contribution. If these conditions are met, nuclear and cytosolic Ca^2+^ will oscillate with clearly independent frequencies, see Fig. 4. For these conditions to be met, coupling strength must be strong enough: Additional file 2 presents the consequences of weaker couplings.

For completeness, we have further explored some situations that promote simultaneous nuclear/cytosolic Ca^2+^ release. One way to couple and try to synchronise nuclear and cytosolic refractory periods is to make their duration depend solely on the rate of decrease of the actual local Ca^2+^ concentration. In case pores were able to homogenise calcium concentrations on either side of the NE in between firings, the nuclear and cytosolic channels could then fire simultaneously. Otherwise, cytosolic and nucleosolic channels would still fire at different times, but their firings times would not be autonomous, see Additional file 2.

Note on parameters: When considering a realistic number of pores, the simulation of Eq. 9 becomes very computationally expensive. One way to partially overcome this problem is to choose parameters that lead to small refractory periods, in order to investigate several calcium oscillation cycles in a shorter simulation time. The relative magnitude of the refractory period does not interfere with the interaction between nuclear and cytosolic Ca^2+^ oscillations, which is the object of our interest.

In the main text, we choose the fraction *α* of the threshold as *α*=0.15. There, we were considering the Ca^2+^ levels at an isolated channel, as explained in this subsection. The important point is that the actual local Ca^2+^ levels, which include the contribution of other channels and pores, do not drop below the activation threshold, at least for some channels. Otherwise the continuation of the CICR mechanism would require some external stimulus.

### Notes on the 2D approximation

By restricting ourselves to a 2 dimensional model of the surfaces, we can dramatically increase the efficiency of our simulation. Since the key components involved in perinuclear signalling, the pumps, channels and pores, are localised to the nuclear membrane, the inner and outer nuclear surfaces are the most important regions to consider when modelling signal generation. However, diffusion of Ca^2+^ away from the surface is an important consideration.

Extending the FDF model, Pando et al. [73] investigated whether the diffusion into the bulk of calcium released from surface sources, would have any effect on the successive activation of calcium channels. He shows that in the continuum limit, the velocity of the calcium wave becomes independent of the diffusion coefficient, *D*, as *D* increases, and then it fails to propagate when *D* is large enough. This counterintuitive effect is due to the finite nature of the calcium source in the continuum limit.

For diffusion spreading from calcium channels in the saltatory regime Eq. 2 considered in our paper, the effects of large *D* would not be observed even for unrealistically large *D*. For the pores sources, the regime is not so clear: pores are more closely spaced than channels and release calcium until the concentration gradient vanishes. However, the ability for pores to mediate efficient signalling should remain. This ability depends on a diffusion path from the channels in the surface with larger *D*, via pores, to channels on the other side with smaller *D*. The first leg of the path (from channels to pores, on the surface with larger *D*) is not likely to fail due to the saltatory regime for the calcium source. The second leg (from pores to channels on the surface with small *D*), isn’t likely to fail either even if sources - pores - would operate in the continuum limit, because *D* is small in this surface.

Moreover, even if we had considered a continuum wave limit, it is not clear whether a similar effect would be acting in our system. In Pando et al. [73], diffusion into the bulk acts as a sink, which is more efficient the larger *D* is. However, they don’t count the effect of the pumping rate as a sink, and as we saw (Eq. 6), pumps are less effective the larger is *D*. This is not likely to change in a 3D context.

Additionally, propagation into a cell or a nucleus would not make calcium vanish into an infinite space. Particularly on the inner nuclear membrane where paths between two points through the bulk are shorter than paths along the surface.

To model the complex geometry of the perinuclear signalling process is beyond the scope of this work. In biological systems, diffusion is typically anisotropic, with diffusion coefficients in the bulk and cellular membranes varying significantly. Additionally, cellular environments are crowded and we don’t expect free diffusion over long distances. Nevertheless, the extension of our model to a 3D geometry would be a very important future development. As the results of Pando et al. [73] showed, bulk diffusion may possibly lead to unexpected results. Moreover, nuclear Ca^2+^ signals can be modulated by the morphology of the nucleus [72].

### Abbreviations

CICR, calcium-induced calcium release; FDF, fire-diffuse-fire model; IP_3_, inositol 1,4,5-trisphosphate; IP_3_R, inositol 1,4,5-trisphosphate receptors; NE, nuclear envelope

## Additional files

Additional file 1 CICR follows a diffusion path integrating all channels and pores. Contains a figure that illustrates how an alternative diffusion path requires the existence of pores and channels on both sides of the NE. (PDF 176 kb)

Additional file 2 Notes on the effect of the refractory period on Ca^2+^ signatures. Contains a discussion and figures concerning an alternative implementation of an refractory period, and also the effects of coupling strength on the similarity between nuclear and cytosolic calcium signatures. (PDF 804 kb)

Additional file 3 Modelling calcium diffusion on the nuclear surface. Contains a comparison between the solutions for the diffusion equation in a surface without boundaries - the approximation used in this paper - and the solution in a surface with periodic boundary conditions. (PDF 236 kb)

Additional file 4 Critical surface for propagation failure. Contains a figure that illustrates how a small diffusion constant and large uptake rate can lead to propagation failure, as explained in Methods subsection Conditions for signalling propagation on an isolated membrane. (PDF 1590 kb)
