# Supplementary material for: Nuclear pores enable sustained perinuclear calcium oscillations
Source: BMC Syst Biol. 2016 Jul 22;10:55. doi: 10.1186/s12918-016-0289-9 (PMC4957432; doi:10.1186/s12918-016-0289-9)
Supplement: Additional file 1 — CICR follows a diffusion path integrating all channels and pores. Contains a figure that illustrates how an alternative diffusion path requires the existence of pores and channels on both sides of the NE. (PDF 176 kb) [file 12918_2016_289_MOESM1_ESM.pdf]

# Additional File 1: CICR follows a diffusion path integrating all channels and pores

We investigated the impact of pores on the propagation of $Ca^{2+}$ signals on either side of the NE, considering calcium release channels on both sides of the NE, Fig 1. We studied the release of $Ca^{2+}$ from a channel, $N_1$, and the activation of another channel, $N_2$, on the nucleosolic side of the NE. We incorporated channels, $C_1$ and $C_2$, on the cytosolic side of the NE. The propagation of a $Ca^{2+}$ burst across both sides of the NE for different pore distributions and a given channel arrangement is shown in Fig 1. As in the main text, the inner nuclear membrane is characterised by a lower diffusion constant $D$. Pores clustered around the channels $C_1$ and $C_2$ are located far from the diffusion range of the channel $N_1$, which results in the firing of $N_1$ being unable to trigger further release events, Fig 1A (crossed beige arrow). However, when pores were clustered around channels $N_1$ and $N_2$ the situation changed. In Fig 1B the arrows follow $Ca^{2+}$ propagation from its release event in $N_1$ as it crosses the NE through the nearby pore to activate channel $C_1$ where it spreads to activate $C_2$, reenters the nucleoplasm and finally triggers the firing of channel $N_2$.

It is the presence of $Ca^{2+}$ release units on both sides of the NE that enables alternative paths, from channel to channel through nuclear pores, to be exploited. Diffusion across the NE would be expected to equilibrate $Ca^{2+}$ concentration on both sides of the NE, thus driving $Ca^{2+}$ transmission to a halt. Moreover, by doubling the space that $Ca^{2+}$ can potentially diffuse in, a permeable nuclear envelope could reduce the local $Ca^{2+}$ concentration, preventing CICR activation. Channels $C_1$ and $C_2$, however, act as $Ca^{2+}$ amplifying units and allow for concentrations differences to be maintained. The arising concentration differences drive the reentry of $Ca^{2+}$ back into the nucleus and trigger the firing of the second nuclear channel $N_2$, as shown in

Fig 1B.

Thus, pores may contribute to the robustness of $Ca^{2+}$ signalling and allow for firing to proceed in conditions where CICR would otherwise fail. This also suggests a reason for the existence of signalling machinery to be on both sides of the NE, such as during plant symbiosis [1, 2] where all the $Ca^{2+}$ decoding elements seem to be located inside the nucleus and the existence of a $Ca^{2+}$ signalling machinery on the outer side of the membrane may seem superfluous. This coupled signalling machinery might offer an advantage to cells that need to maintain oscillations in a fluctuating environment for long durations. For instance, during legume symbiosis a minimum number of sustained nuclear $Ca^{2+}$ spikes is required to elicit a response [3]. The role of a $Ca^{2+}$ signalling machinery on the outer nuclear membrane may be to sustain nucleosolic $Ca^{2+}$ oscillations, facilitated by pores, elements of which are essential for nuclear $Ca^{2+}$ signalling during legume symbiosis [4, 5].

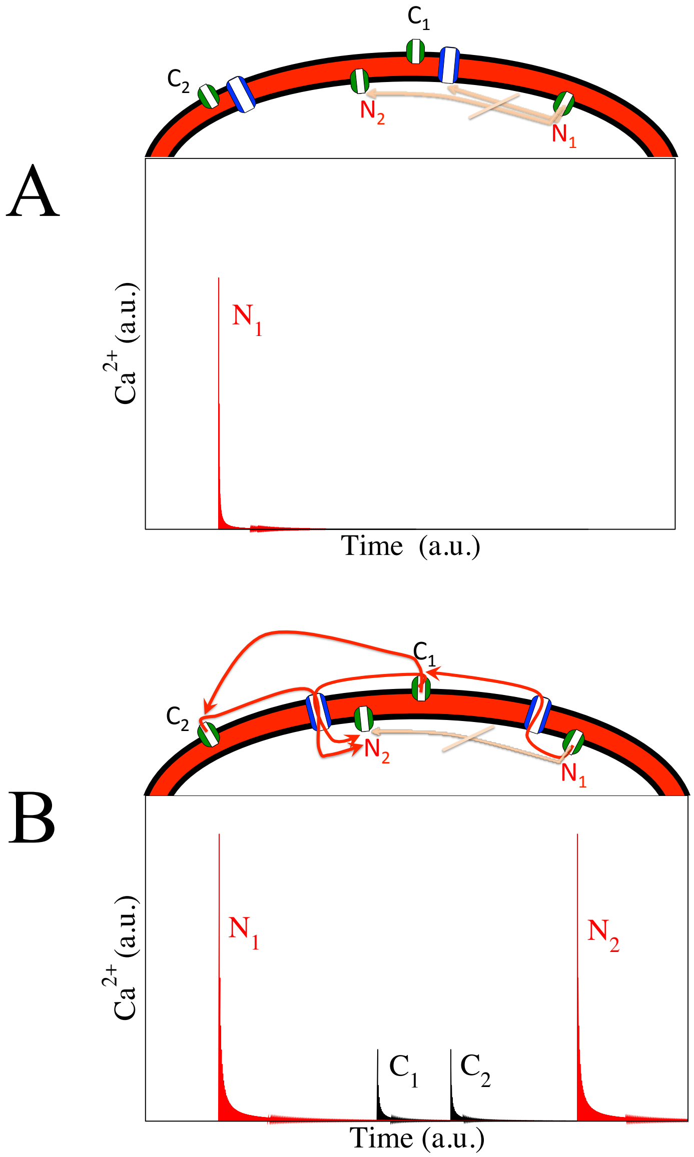


Figure 1: **$Ca^{2+}$ diffusion through pores may create additional activation paths, permitting the exploitation of more favourable propagation conditions on the other side.** We cluster 4 pores around either the outer nuclear membrane (ONM) channels $C_1$ and $C_2$ (upper panel) or the inner nuclear membrane (INM) channels $N_1$ and $N_2$ (lower panel). The simplified scheme illustrates how $Ca^{2+}$ spreads. The crossed beige arrows represent the disruption of $Ca^{2+}$ spread caused by a low diffusion constant, while the red arrows show the spreading of $Ca^{2+}$ as it activates channels leading them to fire in sequence as seen in the main plots that show $Ca^{2+}$ levels surrounding channels (arbitrary units). Diffusion on the nuclear surface is $D_N$=5 $\mu$m$^2$/s, and on the cytosolic side is $D_C$=20 $\mu$m$^2$/s

# Bibliography

[1] Capoen, W., et al.: Nuclear membranes control symbiotic calcium signalling of legumes. Proc. Natl. Acad. Sci. U.S.A **108**(34), 14348–14353 (2011)

[2] Oldroyd, G., Downie, J.: Nuclear calcium changes at the core of symbiosis signalling. Curr. Opin. Plant Biol. **9**, 351–357 (2006)

[3] Charpentier, M., Oldroyd, G.E.: Nuclear calcium signaling in plants. Plant Physiology **163**(2), 496–503 (2013)

[4] Saito, K., et al: Nucleoporin85 is required for calcium spiking, fungal and bacterial symbioses, and seed production in lotus japonicus. Plant Cell **19**, 610–624 (2007)

[5] Kanamori, N., et al.: A nucleoporin is required for induction of ca2+ spiking in legume nodule development and essential for rhizobial and fungal symbiosis. Proc Natl Acad Sci USA **103**, 359–364 (2006)
