# Supplementary material for: Nuclear pores enable sustained perinuclear calcium oscillations
Source: BMC Syst Biol. 2016 Jul 22;10:55. doi: 10.1186/s12918-016-0289-9 (PMC4957432; doi:10.1186/s12918-016-0289-9)
Supplement: Additional file 2 — Notes on the effect of the refractory period on Ca2+ signatures. Contains a discussion and figures concerning an alternative implementation of an refractory period, and also the effects of coupling strength on the similarity between nuclear and cytosolic calcium signatures. (PDF 804 kb) [file 12918_2016_289_MOESM2_ESM.pdf]

Additional File 2: Notes on the effect of the refractory period on Ca^{2+} signatures

The CICR activation of channels could result in an endless Ca^{2+} release from a single site, if not for the imposition of a minimum resting time between successive firings from a single channel [1]. This refractory period imposes a maximum firing rate for an individual channel, and it affects the aggregated channels' firing frequency.

In this file, we explore the combined impact of the choice of the refractory period and coupling strength on the similarity between nuclear and cytosolic Ca^{2+} oscillations. In all the cases considered in this file, to aid in simulating the effect of the coupling strength over a large number of time steps, we use a simplified model that uses the fact that only a few pores contribute significantly to the transmission of Ca^{2+} between channels on different sides of the NE. Considering 24 channels on one side of the NE as usual, and a given number of evenly spaced pores, we pre-compute the minimum distance d_{min} between a channel and a pore. We then associate with each channel a single pore at a distance d_{min}. In case one side has a significantly lower diffusion constant, the pores near the channels on that side will have a much larger impact, so on a first approximation we can neglect the contribution of other pores. To investigate the impact of pore clustering around different positions, we can place the pores near channels on either side. As a further simplification, we neglect the interaction between the n_p pores, avoiding n_p^2 loops. For the interaction between two channels, we consider that there is a pore at distance d_{min} from one channel, on the great-circle joining them. To account for the fact that the nearest pore contributes the most, but others also have a small contribution, we can multiply the contribution of this single pore by a scaling factor (e.g. 6, for the results in this additional file).

In what follows, we will consider conditions in which the inner nuclear membrane (INM) cannot sustain autonomous Ca^{2+} oscillations, whilst the outer nuclear membrane (ONM) can. To visualise the times of Ca^{2+} release, we will show the Ca^{2+} concentrations at channels locations. As in the main text, we note that although inner and outer channels release the same amount of Ca^{2+}, the microdomains of Ca^{2+} concentration persist for a longer time near the channels localised on the membrane with smaller diffusion constant D. Therefore, an easy way to identify the channels on that membrane is to look for the larger amplitudes in the figures below: in this case, the red lines (INM) have larger values than the black ones (ONM).

0.1 Coupling may synchronise Ca^{2+} oscillations if the channels' refractory periods are the same

As seen in Fig. 4A1 and 4B1, of the main text, the time delay of transmission of the first Ca^{2+} wave is negligible and the duration of the nuclear and cytosolic waves is similar. This implies that if activation was the only requirement for the channels' release, nucleosolic and cytosolic Ca^{2+} oscillations would be synchronised.

That is the situation illustrated in Fig. 1, where the red lines depict the temporal evolution of the Ca^{2+} concentration around the nucleosolic channels, localised in a membrane with low D =2 μm^2/s that prevents autonomous sustained Ca^{2+} release. The black lines correspond to the cytosolic channels, which, in both panels, are localised in a membrane with larger D =20 μm^2/s: the same in both panels. In both panels the refractory periods are the same for channels in the inner or outer nuclear membrane. The different panels correspond to different imposed refractory periods: 3.3 seconds (Fig. 1A) versus 2.4 seconds (Fig. 1B). The peaks of the oscillations are synchronised, with the caveat that each nucleosolic cycle of oscillations is broader than the corresponding cytosolic one, because the diffusion part of the fire-diffuse-fire process occurs slowly on a membrane with low diffusion coefficient D.

For this result, Ca^{2+} transmitted by pores needs to be amplified by nuclear channels, which were the missing element in the mathematical models reviewed in the Introduction: those models could not reproduce similar Ca^{2+} transients purely by passive diffusion, as they lacked release channels on both sides.

0.2 Passive diffusion may or may not synchronise the refractory periods if they depend solely on Ca^{2+}

Ca^{2+} channels are activated at small Ca^{2+} levels and then inactivated when Ca^{2+} levels increase further following Ca^{2+} release [2]. A possible option is to assume that the refractory period depends solely Ca^{2+} levels. The channels become ready to fire again when the local Ca^{2+} levels drop to a certain fraction, α, of the threshold, assumed to be the same for both sides.

This approach is illustrated in Fig 2. On an isolated inner nuclear membrane, we make a nucleosolic channel fire at start, Fig 2A, and this same channel can fire again when its local Ca^{2+} levels decrease below a fraction α=1.1 of the activation threshold. Since $\alpha > 1$, the calcium levels are always above the activation threshold, so Fig 2A illustrates the firing of the channel at its maximal allowed rate. Nevertheless, the diffusion constant for Ca^{2+} on this membrane is so low - D_{red}=2 μm^2/s -, that the initial firing channel can not activate any other nucleosolic channel.

If we couple the membranes, Figs 2B and C, cytosolic Ca^{2+} release enables the firing of additional nucleosolic channels.

Since the diffusion coefficients are different, nuclear and cytosolic Ca^{2+} levels decrease at different rates, resulting in different firing frequencies on the two sides of the NE. However, we can see that the frequencies are not autonomous: for instance, now a nucleosolic channel fires less often than in the isolated membrane - calcium levels drop more slowly - but since different nucleosolic channels are firing at different times, the intervals where not a single nucleosolic

channel is firing are shorter than what was observed in Fig 2A. In fact, what we call the overall frequency in Fig 2A is determined by a single firing channel, the initial firing one, whilst in the other cases there are several channels firing at different times, but each channels fires less frequently than in Fig 2A.

However, if the levels that Ca^{2+} needs to drop to were low enough to give pores time to homogenise concentrations inside and outside the NE, in between firings, nuclear and cytosolic Ca^{2+} release would be expected to synchronise. We did not pursue this definition of refractory period in the main text because, although it would provide a way to account for coupled refractory periods within the FDF framework, as it was not appropriate for the question we were asking. Instead we tested the implications of different hypotheses: of either equal or different refractory periods.

We note that for this implementation of the refractory period we need to choose the fraction of the threshold that Ca^{2+} needs to decrease to as $\alpha > 1$, so that the Ca^{2+} concentration remains above the activation threshold. Otherwise, we would need to introduce an external Ca^{2+} stimuli to activate the channels whenever they emerged from their refractory period.

0.3 Refractory period depends on calcium levels and on the characteristics of the channels

Alternatively, as in the main text, we could equate the refractory period to the time taken for the concentration of Ca^{2+} at an isolated source to fall below αc_{th} after it has fired. In this case, channels on different sides of the nuclear membrane will have different maximum firing rates. It is this approach we took in the main paper: it allows us to acknowledge the unknown factors responsible for the duration of the refractory period, besides Ca^{2+}, and by uncoupling the individual refractory periods, it is a suitable option to ascertain if the pores can play any role beyond synchronising oscillations.

In this case, as shown in Fig 4 in the main text, a transparent NE is unable to synchronise nuclear and cytosolic oscillations. Both sides seem to oscillate robustly with their own frequency. It could be expected that if a transparent NE cannot synchronise oscillations, a weaker coupling would definitely not. But in what follows, we show this is not the case. To investigate how coupling strength affects the interaction between the Ca^{2+} signatures, we considered two scenarios of increasing nuclear Ca^{2+} influx, for which we take a higher diffusion on the cytosolic side of the NE.

We observe that autonomous oscillations cannot be sustained if nuclear Ca^{2+} is strongly buffered (low diffusion constant D), Fig. 3A. We then couple both sides of the NE by placing a single pore near each channel either on the side with larger or lower diffusivity, Fig. 3B and C, respectively. The NE is not transparent in either scenario, but Ca^{2+} flow is large enough to trigger the activation of channels across the NE. Upon crossing the NE to activate a cytosolic channel, Ca^{2+} reenters the nucleus giving rise to oscillations on both sides of the NE. Therefore, flow through pores is essential sustained calcium release from channels on a surface where CICR would fail. But the effects on the signatures of the driven channels depend on the coupling strength.

The results shown in Fig. 3B are for a very weak coupling between the cytosolic and nucleosolic compartments, created by placing the pore near the channel on the side with larger D. Since the two sides are weakly coupled, nucleosolic channels can only be activated at the same time that Ca^{2+} flow increases due to a cytosolic Ca^{2+} wave. In this sense, the cytosolic side clearly imposes a firing pace, making the nuclear channels fire at a lower rate than the maximum allowed by their refractory period. However, the larger refractory period of the nucleosolic channels prevents them from firing every time there is a cytosolic wave. Nucleosolic channels fire half as frequently as the cytosolic channels, retaining a partial autonomous frequency. In these conditions where the nucleosolic channels have to wait until cytosolic channels are firing,

if we had decided that the weaker surface would have a faster upper firing rate, the nuclear and cytosolic oscillations could arguably become synchronised.

Fig. 3C considers a still weak but stronger coupling, achieved by placing the pore near the channel on the side with smaller D. As in the example illustrated in Fig. 3B, the first cytosolic Ca^{2+} wave is not sufficient to activate all the nuclear channels. However, coupling is now strong enough to activate additional nucleosolic channels, when Ca^{2+} flow increases due to subsequent cytosolic Ca^{2+} waves. This leads to repetitive firings from the inner nuclear membrane. Therefore, when we increased the coupling strength, we cannot identify a clear Ca^{2+} oscillation frequency on the side with lower diffusion constant.

And as we saw, when the NE is transparent (as in the main text, Fig 4), the firing frequency is autonomous, even though the persistence of firings on one side depends on the calcium flow from the other side. Surprisingly, nuclear and cytosolic signatures reveal the strongest hints of their coupling, exactly when the coupling is the weakest. A transparent NE allows sufficient Ca^{2+} between membranes to immediately activate channels the moment they drop out of their refractory period. Weakest coupling, Fig. 3B, only activates a fraction of Ca^{2+} channels and they need to wait for a fresh cytosolic wave to be activated again. The less weak but not transparent coupling, Fig. 3C, corresponds to an intermediate case where different nucleosolic channels are experiencing different levels of Ca^{2+} and are firing in a more complicated unsynchronous way.

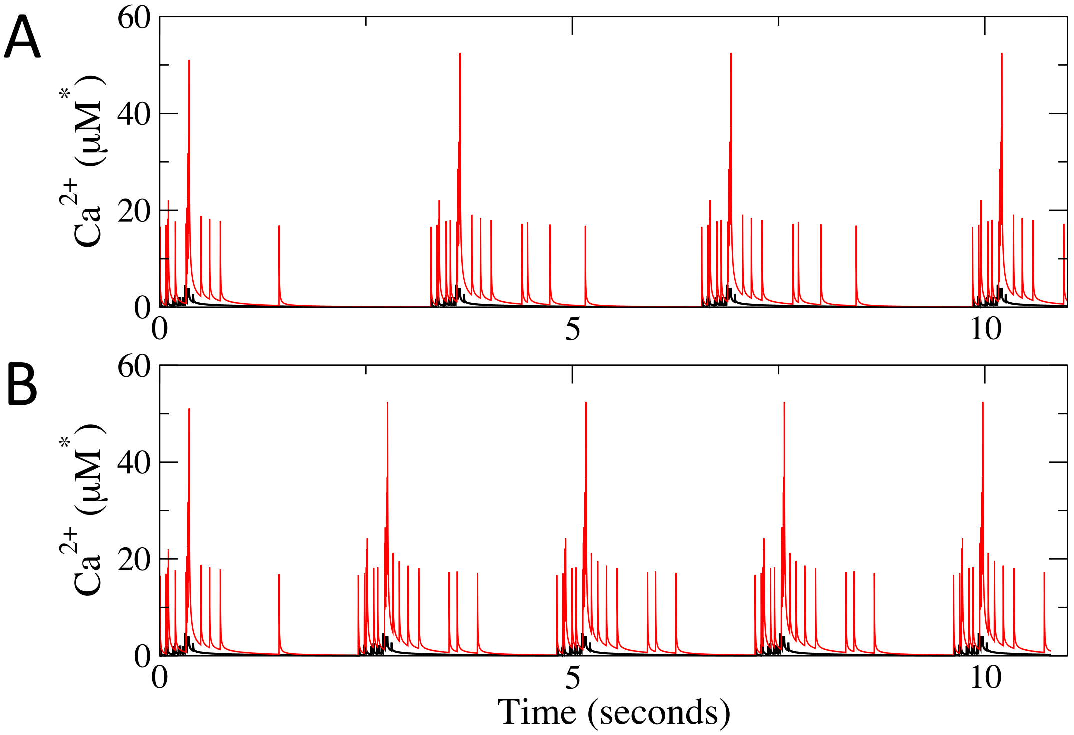

Figure 1: **Passive diffusion through pores can synchronise release events from distinct membranes if refractory period are the same** D_{red}=2 μm^2/s: channels on this membrane would not would be able to sustain autonomous Ca^{2+} release. Oscillations are sustained when coupled to channels on a membrane where D_{black}=20 μm^2/s. The different panels correspond to different imposed minimum resting interval between firings: 3.3 seconds (A) versus 2.4 sec (B). Parameters: c_{th}=0.020 μM*, α=0.15, k_s=1 s^{-1}. The use of M* units indicates that it is not strictly a volumetric concentration, see the first subsection of Methods.

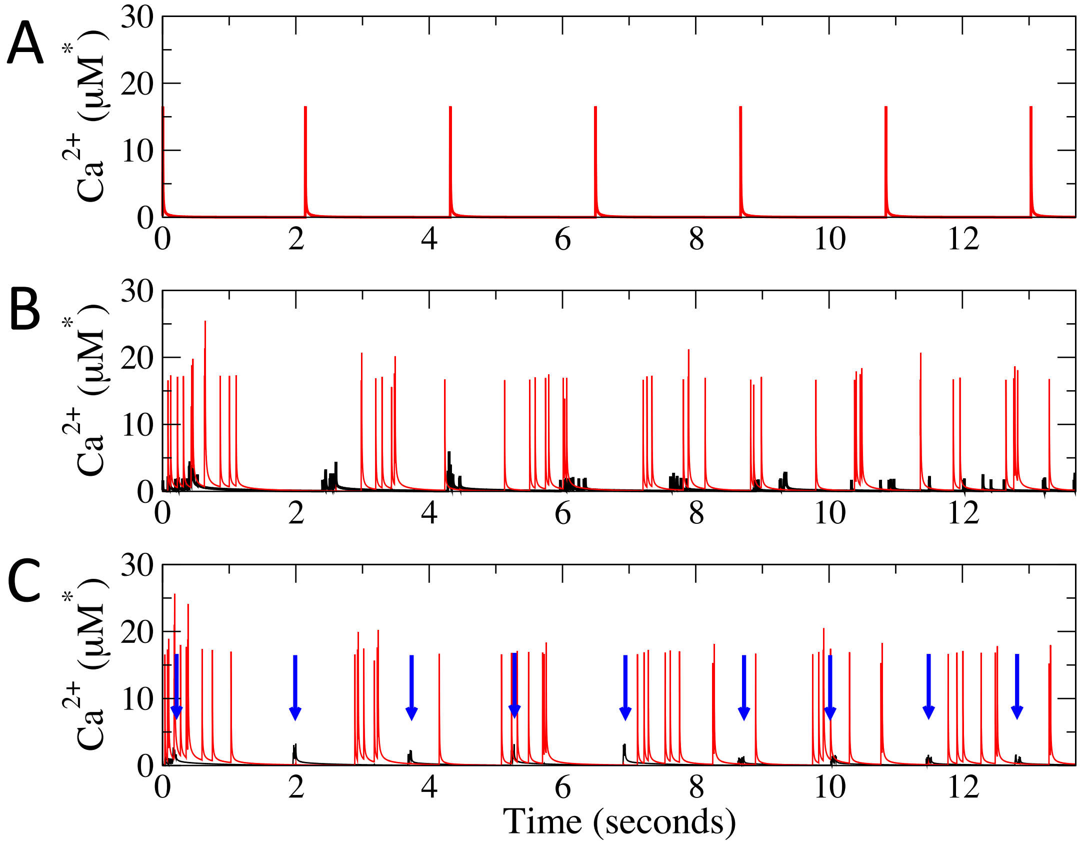

Figure 2: **Passive diffusion through the pores synchronises some but not all of the release events if refractory period is determined solely by Ca^{2+} concentration at the channel.** Panel A: isolated membrane with D_{red}=2 μm^2/s. Other panels: coupling with a membrane with D_{black}=20 μm^2/s (panel B) or D_{black}=50 μm^2/s (Panel C). To improve clarity, the blue arrows in panel C indicate the times when the cytosolic channels are firing. Parameters:α=1.1, random locations, k_s=1 s^{-1}. The use of M* units indicates that it is not strictly a volumetric concentration, see the first subsection of Methods.

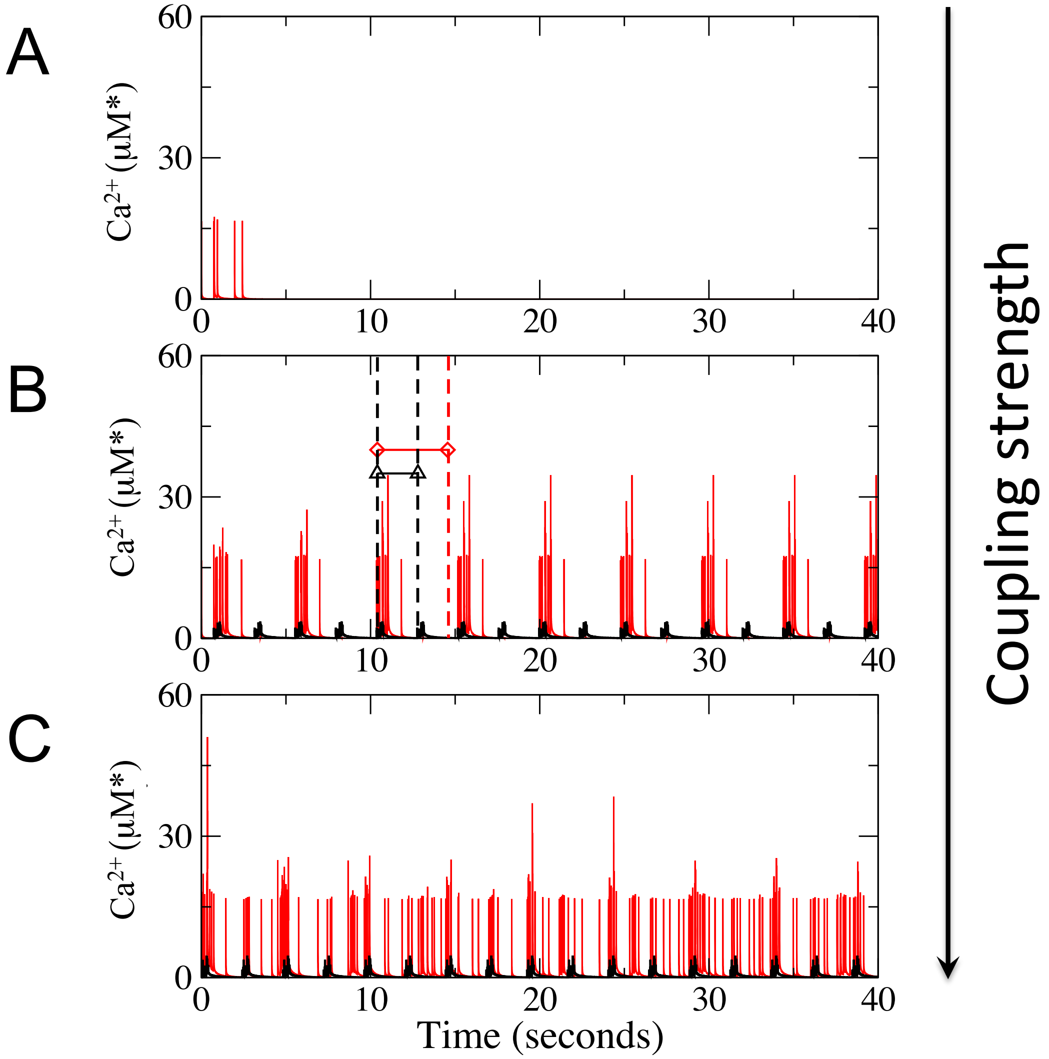

Figure 3: **Clustering of pores around channels on different surfaces result in different Ca^{2+} signatures.** Ca^{2+} profiles on the INM with D=2 $\mu m^2/s$ (red lines), and on the ONM with D=20 $\mu m^2/s$ (black lines). There are 24 channels placed randomly on each side. (A) Even if the INM has an independent basic signalling machinery exactly equal to the cytosolic side, it is unable to sustain autonomous oscillations if nuclear calcium is strongly buffered (low D), resulting in an interruption of Ca^{2+} release after just a few have fired. (B, C) illustrate how the coordination between calcium release on the inner and outer nuclear membranes depends on whether the pores are near the channels on the side with a low diffusion constant (B) or large diffusion constant (C). The dashed vertical lines in the middle panel B indicate the duration of the refractory period of the channels on the inner (diamond, red) and outer (triangle, black) nuclear membranes. For this figure, rather than simulating all 4024 pores, we use a simplified form of the model described in this document. Other parameters: c_{th}=0.020 μM*, α=0.15, k_s=1 s^{-1}. The use of M* units indicates that it is not strictly a volumetric concentration, see the first subsection of Methods.

Bibliography

[1] Timofeeva, Y., Coombes, S.: Wave bifurcation and propagation failure in a model of ca^{2+} release. J. Math. Biol **47**, 249–269 (2003)

[2] Dawson, S., Keizer, J., Pearson, J.: Fire-diffuse-fire model of dynamics of intracellular calcium waves. Proc. Natl. Acad. Sci. U.S.A **96**(11), 6060–3 (1999)
