# Supplementary material for: Nuclear pores enable sustained perinuclear calcium oscillations
Source: BMC Syst Biol. 2016 Jul 22;10:55. doi: 10.1186/s12918-016-0289-9 (PMC4957432; doi:10.1186/s12918-016-0289-9)
Supplement: Additional file 3 — Modelling calcium diffusion on the nuclear surface. Contains a comparison between the solutions for the diffusion equation in a surface without boundaries - the approximation used in this paper - and the solution in a surface with periodic boundary conditions. (PDF 236 kb) [file 12918_2016_289_MOESM3_ESM.pdf]

Additional File 3: Modelling calcium diffusion on the nuclear surface

0.1 Analytic solution to diffusion equation

As described in the Methods, the calcium concentration, $c(\mathbf{r}, t)$, on the surface of the nucleus evolves as

$$\frac{\partial c}{\partial t} = D\nabla^2 c - k_s c + \sigma \sum_{i=1}^{N} \sum_{k=1,2,\ldots} \delta(t - t_i^k)\delta(\mathbf{r} - \mathbf{r}_i), \tag{1}$$

for diffusion constant, D, uptake rate, k_s, and release strength σ. The form of this diffusion equation allows us to derive an analytic solution using Green's method. On the surface of a sphere of radius, R, Green's function satisfies

$$\frac{\partial G}{\partial t} = D\nabla^2 G - k_s G + \frac{1}{R^2 \sin\theta'}\delta(\theta - \theta')\delta(t - t'), \tag{2a}$$

$$G(r, \theta, t | r', \theta', t') = 0, t < t' \tag{2b}$$

where, thanks to the symmetry, we can neglect the ϕ dependence for now, including it later via trigonometric properties [1]. The resulting calcium concentration for the given source term (1) is,

$$c(\mathbf{r}, t) = \sigma \sum_{k=0}^{N} G(\mathbf{r}, t | \mathbf{r}_k, t_k). \tag{3}$$

We shall now derive an exact expression for the Green's function which will enable us to evaluate the behaviour of the calcium on the surface in a numerically efficient way. We follow the approach of Skupin *et al.* [1]. After Laplace transform with respect to t, the governing equation of the transformed Green's function $\tilde{G}(\theta, s|\theta', t')$ is

$$s\tilde{G} = D\nabla^2 \tilde{G} - k_s \tilde{G} + \frac{1}{R^2 \sin\theta'}\delta(\theta - \theta')e^{-st'}. \tag{4}$$

The homogeneous problem

$$\frac{1}{R^2 \sin\theta}\frac{\partial}{\partial\theta}\left[\sin\theta\frac{\partial\psi}{\partial\theta}\right] = -\frac{l(l+1)}{R^2}\psi, \tag{5}$$

is the well known Legendre differential equation, whose solution are the Legendre polynomials

$$\psi_l(\theta) = P_l(\cos\theta). \quad l = 0, 1, 2, \ldots \tag{6}$$

We then consider the ansatz

$$\tilde{G}(\theta, s|\theta', t') = \sum_{l,p=0}^{\infty} \beta_l \psi_l(\theta), \tag{7}$$

which, upon substitution into (4), yields

$$s\sum_{l=0}^{\infty}\beta_l P_l(\cos\theta) = -D\sum_{l=0}^{\infty}\beta_l\frac{l(l+1)}{R^2}P_l(\cos\theta) - k_s\sum_{l=0}^{\infty}\beta_l P_l(\cos\theta) + \frac{1}{R^2 \mathrm{sin}\theta'}\delta(\theta-\theta')e^{-st'}. \tag{8}$$

To determine the normalisation β_l, we apply the integral operator

$$\int_{-1}^{+1} d\mu P_m(\mu) \tag{9}$$

to obtain

$$s\beta_m = -\beta_m\frac{l(l+1)}{R^2}D - k_s\beta_l + \frac{1}{R^2\mathcal{N}(m)}P_l(\cos\theta')e^{-st'}, \tag{10}$$

where

$$\mathcal{N}(l) = \int_{-1}^{+1} d\mu P_l^2(\mu) = \frac{2}{2l+1}. \tag{11}$$

Using (10) to find β_l, we find the solution in Laplace space

$$\tilde{G}(\theta, s|\theta', t') = \sum_{l=0}^{\infty}\frac{1}{\mathcal{N}(l)(s + Dl(l+1)/R^2 + k_s)}P_l(\cos\theta')e^{-st'}P_l(\cos\theta). \tag{12}$$

This can be transformed back using the inverse Laplace transform and the residue theorem, since we have first order poles, $s + Dl(l+1)/R^2 + k_s$ along the negative axis only. The Green's function solution to the inhomogeneous problem (2a) without the ϕ dependence is

$$G(\theta, t|\theta', t') = \sum_{l=0}^{\infty}\frac{1}{R^2\mathcal{N}(l)}P_l(\cos\theta')e^{(l(l+1)D/R^2+k_s)t'}P_l(\cos\theta)e^{-(l(l+1)D/R^2+k_s)t}. \tag{13}$$

When we wish to consider multiple sources, the assumption of spherical symmetry is no longer valid and we must reintroduce explicitly the ϕ dependence of the Green's function. This only depends on the cosines of the angles between $P(\mathbf{r})$ and $P(\mathbf{r}')$, and hence we can rotate the coordinate system such that one of the angles is zero. The angle Θ between the points is given by

$$\cos\Theta = \cos\theta\cos\theta' + \sin\theta\sin\theta'\cos(\phi-\phi'). \tag{14}$$

The final form of the Green's function is therefore

$$G(\theta,\phi,t|\theta',\phi',t') = \sum_{l=0}^{\infty}\frac{P_l(\cos\Theta)}{2\pi R^2\mathcal{N}(l)}e^{-l(l+1)D(t-t')/R^2-k_s(t-t')}, \tag{15}$$

and the corresponding concentration due to a set of sources at $\mathbf{r}_i$, $i=1,\ldots,N$ firing at times t_k^i for the ith location is

$$c(\theta,\phi,t) = \sigma\sum_{i,k}\sum_{l=0}^{\infty}\frac{P_l(\cos\Theta)}{2\pi R^2\mathcal{N}(l)}e^{-l(l+1)D(t-t_k^i)/R^2-k_s(t-t_k^i)}. \tag{16}$$

$$\cos\Theta = \cos\theta\cos\theta_i + \sin\theta\sin\theta_i\cos(\phi-\phi_i) \tag{17}$$

0.2 Free space Green's function as an approximation to the full solution

Equation (15) is the mathematically precise solution, however evaluating it numerically could be a computationally expensive procedure if a large number of sources are considered (with a large number of pores, for example). It is likely that the dynamics of diffusing calcium on the nuclear surface are such that the dominant contribution from a single source at some point of interest (another channel or the point corresponding to an image pixel say) will be due to calcium that has performed less than one journey around the surface. That is, although strictly speaking the sphere has periodic boundary conditions, on the time scales of interest diffusion would not have been sufficient to allow the periodicity to have an observable effect. It might

be reasonable then to treat the surface as being without a boundary. On the surface of a sphere then, we believe the concentration due to sources at $\mathbf{r}_i$ for $i = 1, \ldots, N$ firing at times t_k^i at the ith location to be well approximated by

$$c(\mathbf{r}_j, t) = \sigma \sum_{i,k} \frac{1}{4\pi D(t - t_k^i)} \exp(-\Delta\mathbf{r}_{ij}/4D(t - t_k^i) - k_s(t - t_k^i)), \tag{18}$$

where $\Delta\mathbf{r}_{ij}$ is, as described in the main text, the length of the great arc connecting the points $\mathbf{r}_i$ and $\mathbf{r}_j$ on the surface of the nucleus. In the long time limit, this gives a vanishing calcium concentration regardless of whether an uptake rate k_s is included, but since we always consider pumps to be present this will not become an issue. In Figure 1A we compare (18) with (16) by calculating the calcium profile for a single point source at $\phi = 0$, for a number of time points. This clearly shows that any difference between the full solution (16; points) and the approximate solution (18; lines) is small. Figure 1B calculates the ratio of the full solution to the approximate solution at the source where the error is largest, for a variety of diffusion constants. While the relative error grows with time, and with larger diffusion constants, the absolute amplitude falls much faster, preventing this effect from becoming a substantial issue. As can be seen in Figures 5 & 6 in the main text, the timescale over which these relative errors grow large is greater than the timescales of oscillations that are studied. Given the approximate solution offers around a 10-fold improvement in the speed of simulation, we are justified in using this simpler model in simulating the surface diffusion of Ca^{2+}.

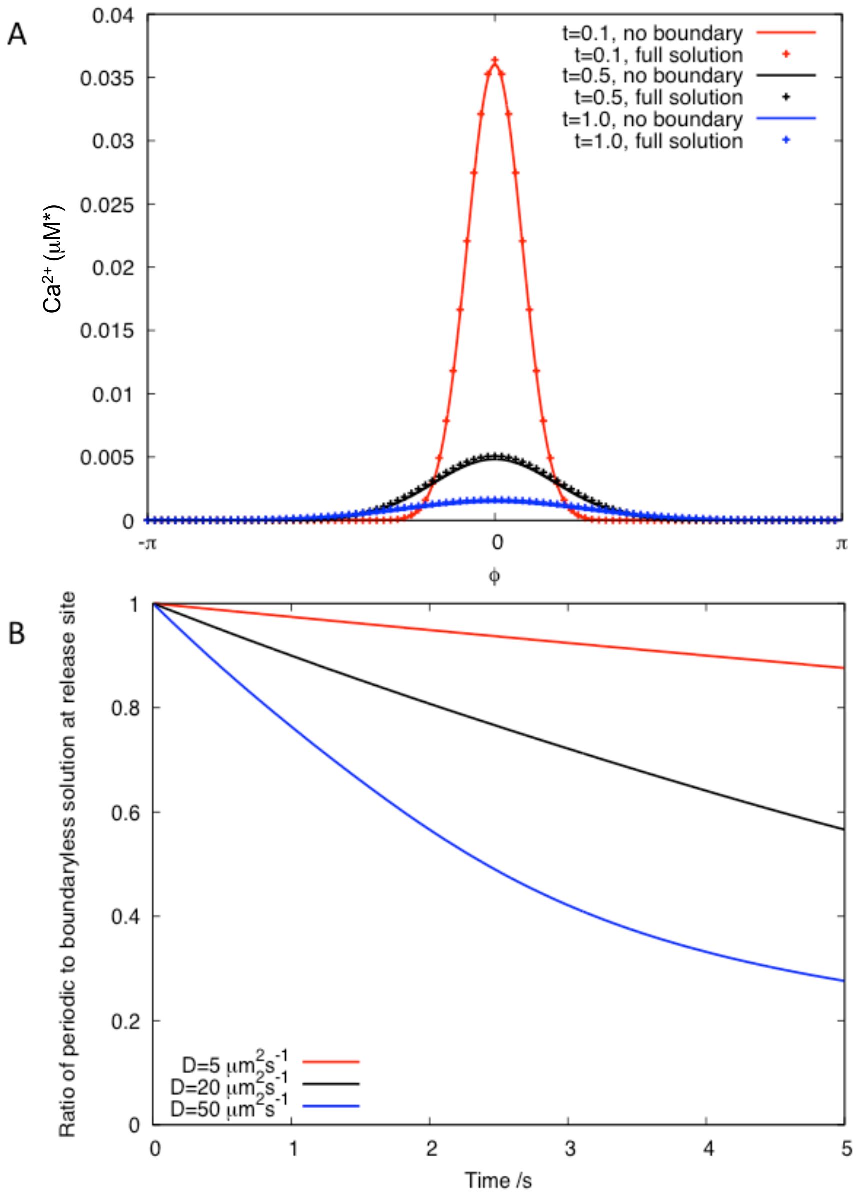

Figure 1: **Boundary free solution provides a good fit to full periodic solution**. Comparing the full periodic solution to the 2D diffusion equation on a spherical surface (16) with the free space solution in 2D (18). (A) Ca^{2+} profiles in cross section ($\theta = \pi/2$). A single channel at $\phi = 0$ releases Ca^{2+} at $t = 0$ and the concentration of Ca^{2+} is calculated at points around the equator of the sphere at different times, $t = 0.1$s (red), $t = 0.5$s (black) and $t = 1.0$s (blue). Lines show the boundary free solution, points are for the periodic solution. Parameters $D = 20$ μm^{2}s^{-1}, $k_s = 1.0$ s^{-1}, $\sigma = 1$. (B) Relative on peak ($\phi = 0$) amplitude in the periodic compared to the boundary free solution against time, and for different diffusion constants. While there can be a large relative shift in amplitude between the two models, as illustrated in (A) the absolute amplitude in both models at these times is very small. The use of M* units indicates that it is not strictly a volumetric concentration, see the first subsection of Methods.

Bibliography

[1] Skupin, A., Kettenmann, H., Falcke, M.: Calcium signals driven by single channel noise. PLoS Computational Biology **6**(8) (2010)
