# Supplementary material for: Nuclear pores enable sustained perinuclear calcium oscillations
Source: BMC Syst Biol. 2016 Jul 22;10:55. doi: 10.1186/s12918-016-0289-9 (PMC4957432; doi:10.1186/s12918-016-0289-9)
Supplement: Additional file 4 — Critical surface for propagation failure. Contains a figure that illustrates how a small diffusion constant and large uptake rate can lead to propagation failure, as explained in Methods subsection Conditions for signalling propagation on an isolated membrane. (PDF 1590 kb) [file 12918_2016_289_MOESM4_ESM.pdf]

Additional File 4: Critical surface for propagation failure

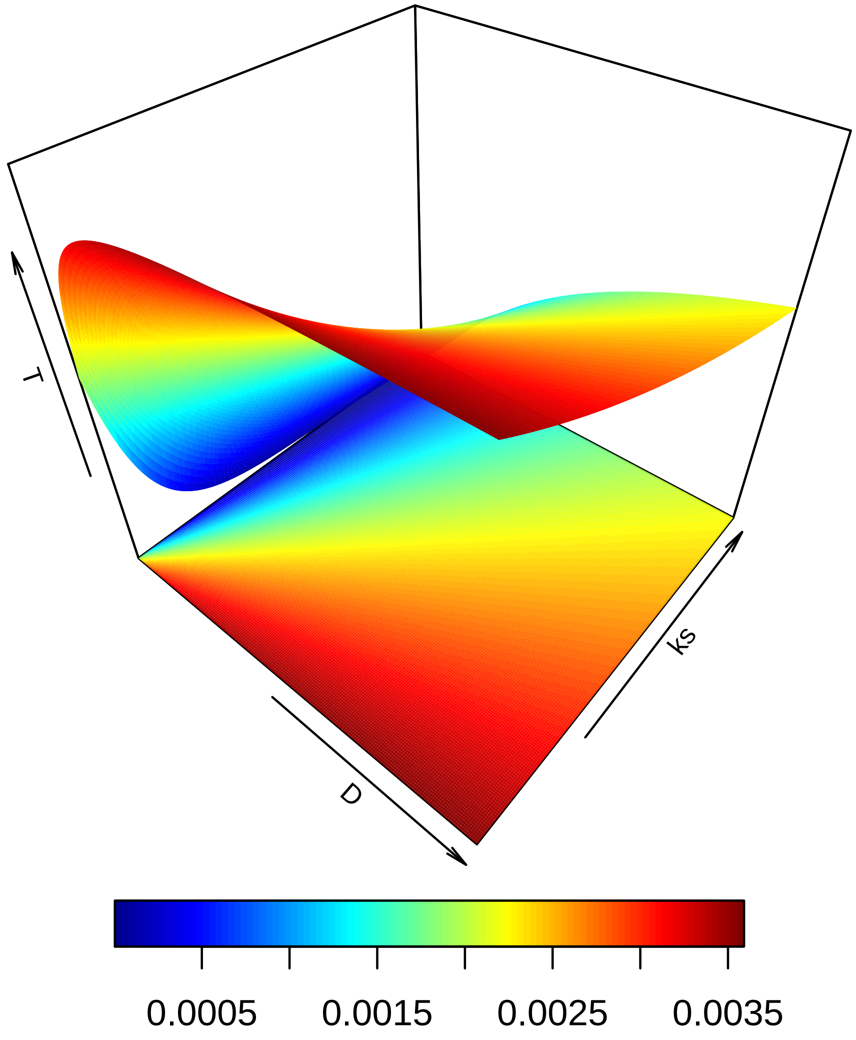

Figure 1: **Ca^{2+} diffusion may fail if D is small or/and k_s is large.** For any D or k_s, if the ratio of the threshold c_{th} to the firing strength σ, defined as T, is above the depicted surface, then a travelling wave will not propagate across the entire surface. Conversely if T is below the surface, a single release event is guaranteed to initiate a propagating wave. Small diffusion constants and large pumping rates lead to propagation failure. In the Figure, D varies between 1 and 40 μm^2/s, and k_s varies between 0.1 and 3 s^{-1}. The average distance between channels is 5.65 μm, corresponding to 24 approximately equidistant channels on a sphere of radius 8 μm.
